# Genome-Wide *cis*-Regulatory Element Based Discovery of Auxin-Responsive Genes in Higher Plant

**DOI:** 10.3390/genes13010024

**Published:** 2021-12-23

**Authors:** Jianfei Wu, Fan Gao, Tongtong Li, Haixia Guo, Li Zhang, Yijie Fan, Aiyun Chen, Jianjun Wang, Fengjuan Shi, Guangyao Shan, Huihui Guo, Fanchang Zeng

**Affiliations:** 1 State Key Laboratory of Crop Biology, Shandong Agricultural University, Tai’an 271018, China; jfwu@sdau.edu.cn (J.W.); fg18325@163.com (F.G.); lttsdau2019@163.com (T.L.); diya_haixiaguo@163.com (H.G.); 15610418001@163.com (L.Z.); yjfan@sdau.edu.cn (Y.F.); aiyunchen11@163.com (A.C.); a1376027271@163.com (J.W.); sfj22432x@163.com (F.S.); perfectguangyao@163.com (G.S.); 2 College of Agronomy, Shandong Agricultural University, Tai’an 271018, China; hhguo@sdau.edu.cn

**Keywords:** auxin-responsive genes, genome-wide discovery, *cis*-regulatory element, *Arabidopsis thaliana*

## Abstract

Auxin has a profound impact on plant physiology and participates in almost all aspects of plant development processes. Auxin exerts profound pleiotropic effects on plant growth and differentiation by regulating the auxin response genes’ expressions. The classical auxin reaction is usually mediated by auxin response factors (ARFs), which bind to the auxin response element (AuxRE) in the promoter region of the target gene. Experiments have generated only a limited number of plant genes with well-characterized functions. It is still unknown how many genes respond to exogenous auxin treatment. An economical and effective method was proposed for the genome-wide discovery of genes responsive to auxin in a model plant, *Arabidopsis thaliana* (*A. thaliana*). Our method relies on *cis*-regulatory-element-based targeted gene finding across different promoters in a genome. We first exploit and analyze auxin-specific *cis*-regulatory elements for the transcription of the target genes, and then identify putative auxin responsive genes whose promoters contain the elements in the collection of over 25,800 promoters in the *A. thaliana* genome. Evaluating our result by comparing with a published database and the literature, we found that this method has an accuracy rate of 65.2% (309/474) for predicting candidate genes responsive to auxin. Chromosome distribution and annotation of the putative auxin-responsive genes predicted here were also mined. The results can markedly decrease the number of identified but merely potential auxin target genes and also provide useful clues for improving the annotation of gene that lack functional information.

## 1. Introduction

The phytohormone auxin, which is ubiquitous in the development of plants, plays a critical role in maintaining plant physiological function and regulating most major plant responses, including cell elongation, division, and differentiation, as well as root initiation, apical dominance, and tropisms responses. Numerous genes that are specifically up/down-regulated by auxin have been described [1,2,3,4,5,6]. These early/primary auxin-responsive genes have been generally grouped into three major categories: GH3, auxin/indoleacetic acid (Aux/IAA), and small auxin-up RNA (SAUR) gene families [3,5,7,8,9].

The classic mode of action of auxin is mediated by a family of *trans*-acting transcription factors that specifically bind to auxin response elements (AuxREs) in the target gene’s promoter region; these factors are known as auxin-response factors (ARFs) [3,4,10,11,12]. A family of auxin regulatory proteins, called Aux/IAA proteins, also play a crucial role in regulating these auxin response genes [13,14]. ARF and Aux/IAA proteins have been supported by substantial experimental evidence to support their role models in regulating early auxin response genes [4,11,15,16,17]. The transcriptional regulation of early responsive genes can be influenced by the types of their interactions with ARFs and Aux/IAAs. Thus far, our understanding is limited to only these two *trans*-acting factors involved. Importantly, ARFs and Aux/IAAs are actually not the only factors participating in the regulation of auxin-responsive transcription. In this process, other essential co-activators and co-inhibitors are necessary. These additional undiscovered transcription factors (e.g., coupling factors) can mediate the targeting to different kinds of composite AuxREs.

The precise mechanism/model of auxin response gene transcription regulation in *A. thaliana* remains to be determined. The signal transduction pathway from auxin perception to gene expression regulation by auxin is undoubtedly complicated. Although information about the mechanism is limited and challenges remain, the eventual effects of auxin are ultimately reflected by the function of auxin-responsive genes. Therefore, identification of auxin-responsive genes is a basic crucial aspect for a comprehensive understanding of auxin’s effects on developmental responses and the physiological function.

Traditionally, hormone auxin research has been based and focused on the studies of individual genes. Latterly, large-scale expression profiling and transcriptome technologies significantly broadened the extension of genes referred in this study. Today, high-throughput and large-scale technologies make it possible to screen a great quantity of auxin-responsive genes. Although these updated technologies can obtain and accumulate a large amount of data, there are still many problems in the research process. For example, a number of genes generated from experiments are lacking well-documented functional information; this large-scale expression data can be a direct or indirect response to auxin treatment, and in-depth exploration of individual auxin-responsive genes is intricate and costly. Thus, it is necessary to develop a rapid, economical, and highly efficient approach to discover plant auxin-responsive genes. This approach can also provide rational clues for those genes for which the functional information is lacking but which may respond to auxin. Thus far, about half of the functional genes in *A. thaliana* are definitively annotated.

A basic problem is screening out genes that respond to specific environmental stimuli in computational genomics research. It can be referred to and is known as “targeted gene finding”. Due to gene regulation being mainly dependent on combining transcription factors with *cis*-regulatory DNA sequences, most current gene annotation methods in existence, which are based on the conservative open reading frames (ORFs), are not sufficient or efficient in finding target genes. In this study, we provide a straightforward, economical, and efficient computing method to predict auxin-responsive gene subclasses with a computer programme called PRECISE (Prediction of Regulatory *CIS*-acting Elements) in the prediction of regulatory elements. Our method aims at detecting functional target genes on a genome scale with minimal false predictions.

Functional information and chromosome distribution maps in the *A. thaliana* genome of the candidate auxin-responsive genes predicted in our study were investigated. Bioinformatic data mining for candidate genes with Gene Ontology (GO), shown to be associated with these sets of putative auxin-responsive genes, were also identified.

## 2. Materials and Methods

### 2.1. Arabidopsis Genomic Promoter Sequences

*A. thaliana* promoter sequences (without fake genes and RNA genes) were collected by combining all *A. thaliana* genomic promoter sequences databases, including the *A. thalianacis*-regulatory database (AtcisDB) (http://arabidopsis.med.ohio-state.edu/, accessed on 10 October 2021), The *A. thaliana* Information Resource (TAIR) (http://www.arabidopsis.org, accessed on 15 July 2021), GenBank (http://www.ncbi.nlm.nih.gov/, accessed on 27 October 2021), and the bioinformatic tool TSSP (SoftBerry, http://www.softberry.com, accessed on 29 October 2021) were used, referencing Zhang et al. (2005) [18]. These tools have been applied to the extraction of promoter sequences.

### 2.2. Prediction of Arabidopsis cis-Acting Regulatory Elements Which Are Responsible for the Transcription of Auxin-Responsive Genes

To predict these *cis*-acting elements, we applied the computer program PRECISE [19]. In this study, thereported available gene collection responsive to auxin induction, which were retrieved as far as possible from the published literature and public transcriptome expression data on *A. thaliana*, were subjected to analysis by PRECISE and compared with the known plant-specific *cis*-elements motifs in the databases of PLACE [20], PlantCARE [21], and TRANSFAC [22] to obtain a degenerate AuxRE for predicting auxin-responsive genes in *Arabidopsis,* as described by Zhang et al. (2005) [18] and Geisler et al. (2006) [23].

### 2.3. Searching for Arabidopsis Genes Containing the AuxRE

The PATMATCH program, a string-search tool that scans the genome-wide upstream sequence in A. thaliana from every annotated gene, was used to identify the genes that had at least one item of the putative AuxRE element in their promoter in A. thaliana. This program and procedure are available on TAIR (http://www.arabidopsis.org, accessed on 15 July 2021), which can generate the identities of genes, number of elements, and position of every element and sequence (where degenerate base codes or wildcards were used).

### 2.4. Evaluation of Candidate Genes

We investigated all major published transcriptome datasets, including GEO and published microarray/omics as described in the following paragraph, to search data evidence for candidate genes that were responsive to auxin treatment. In addition, a literature retrieval was used to determine whether a candidate gene is directly responsive to auxin. We performed a double-evaluation process on the candidate genes. We considered genes as responsive to auxin if they showed >two-fold (either up or down) differential changes of their expression levels after auxin treatment in at least one investigated transcriptome experiment. To search published evidence in the literature, we searched the literature for evidence of candidate genes through the documented experimental evidence literature in the PubMed database for all non-redundant candidate genes.

### 2.5. Transcriptome Data Sources

Transcriptome data were derived from the GEO (Gene Expression Omnibus, https://www.ncbi.nlm.nih.gov/geo/, accessed on 27 October 2021), where more than a hundred auxin-related transcriptomes can be found. Other supplementary public transcriptome databases included TAIR and standardized microarrays from the Nottingham Arabidopsis Science Centre (NASC) (http://affymetrix.arabidopsis.info/narrays/experimentbrowse.pl, accessed on 8 November 2021). Normalization and experimental statistics were performed by the database provider or the original authors.

### 2.6. Chromosome Maps of the Arabidopsis Genome of Auxin-Responsive Genes

We drew chromosome maps of the *A. thaliana* genome of auxin-responsive genes using a list of locus names from the TAIR website (http://www.arabidopsis.org, accessed on 15 July 2021). This tool was useful for mapping many loci of auxin-responsive genes, and we developed a sense of their distributions on *A. thaliana* chromosomes 1–5.

### 2.7. Functional Categories of Candidate Genes

The candidate genes with functional information were defined into two classifications, which were denoted as “confirmed with documented evidence” and “non-confirmed” (unpublished evidence on PubMed). The classification of the confirmed genes applied to those that have been proved to have the direct response to auxin treatment. We assigned genes that did not have any related function information or that produced hypothetical proteins as genes with “function unknown”. The functional diversity of auxin response genes is reflected in the large amounts of functional classifications with which these genes might be involved. We performed general bioinformatic functional annotation analysis on the categories of putative genes set-mined in our research by using the MIPS functional classification from http://mips.gsf.de/projects/plants (accessed on 10 November 2021). GO terms, such as cellular component, biological process and molecular function, can be analysed by using the FatiGO program [24,25,26].

## 3. Results

In our study, we started with the investigation of the *cis*-elements of AuxRE of the auxin-responsive genes in *A. thaliana*. Then we mined candidate target genes and verified them by searching the literature and public datasets related to auxin treatment. This method predicted that 474 candidate genes were responsive to auxin. The 474 genes are considered to be candidate target genes that have an increased possibility of reacting to auxin treatment. We compared our predicted 474 candidate target genes with the transcriptome database and found that 291 (61.4%) of them were responsive to auxin on the basis of a two-fold threshold (Table 1). In addition, we found that public databases did not contain functional annotation information for 198 of 474 candidate genes. For the remaining 276 genes, we found that PubMed contained documented evidence of direct reaction to auxin treatment for 11.6% (32/276). Combining the document search with the analysis from transcriptome databases in which a two-fold threshold was used, we found that 65.2% (309/474) of all 474 candidate target genes are responsive to auxin. Furthermore, if we excluded genes categorized as “function unknown”, the prediction accuracy for the remaining genes was significantly improved, to 76.4% (211/276).

Though, so far, for more than one-third (34.8%) of our candidate genes, no supporting evidence for their response to auxin has been found, future research may reveal that some of the genes possess such functions.

### 3.1. Locations of Auxin-Responsive Genes along the Chromosomes

**Figure 1:**
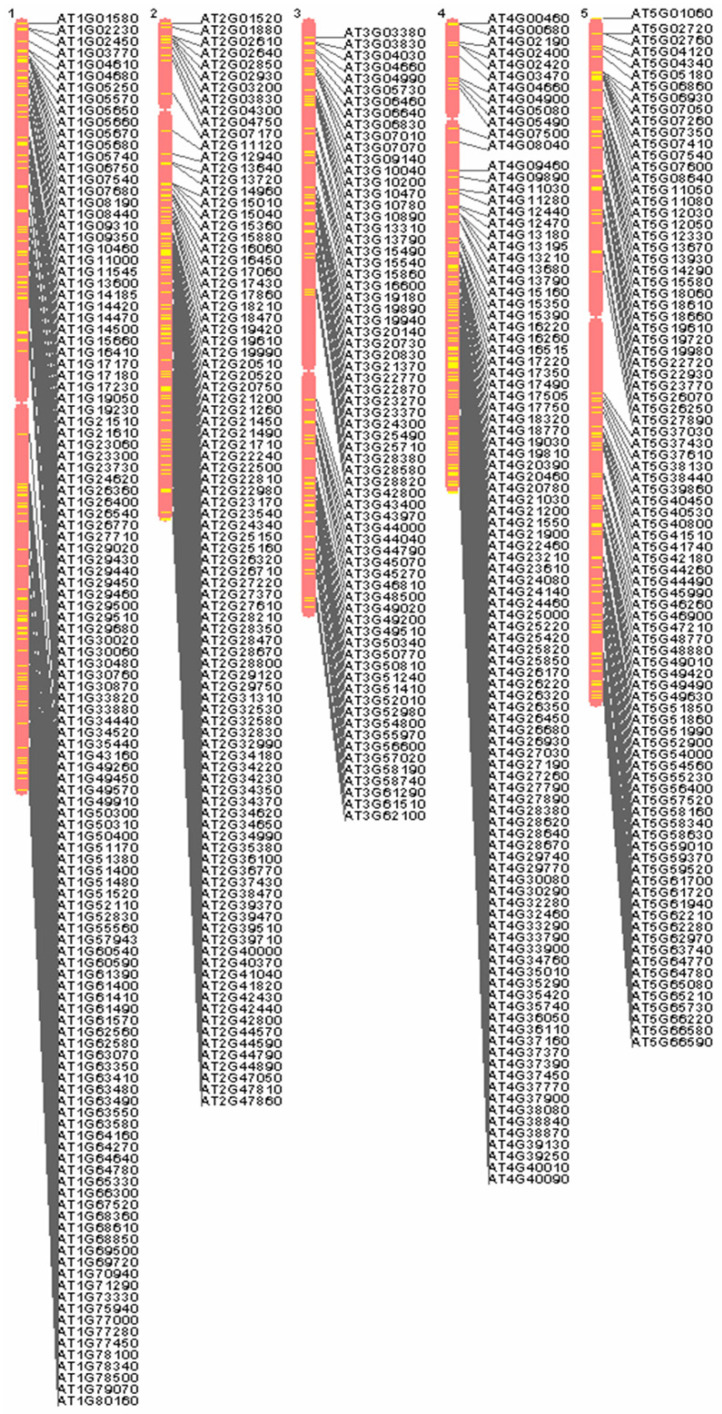
Locations of auxin-responsive genes along the chromosomes based on a list of locus names.

Chromosome maps of the *A. thaliana* genome of auxin-responsive genes were displayed by using a list of locus names (Figure 1) on the *A. thaliana* Information Resource (TAIR) website (http://www.arabidopsis.org, accessed on 15 October 2021). This resource is useful for determining many loci of auxin-responsive genes, and we can get a sense of their distribution on *A. thaliana* chromosomes 1–5. As shown in Figure 1, there is a distinctly different density on *A. thaliana* chromosomes 1–5 (3.8 genes/Mb on average). For example, the shortest chromosome, chromosome 4, contains almost the greatest number of loci of auxin-responsive genes (5.8 genes/Mb). In addition, there is a common feature that few or no loci of auxin-responsive genes are distributed on the centromere of *A. thaliana* chromosomes 1–5.

### 3.2. Biolocigal Process Categories and Data Mining for Candidate Genes

**Figure 2:**
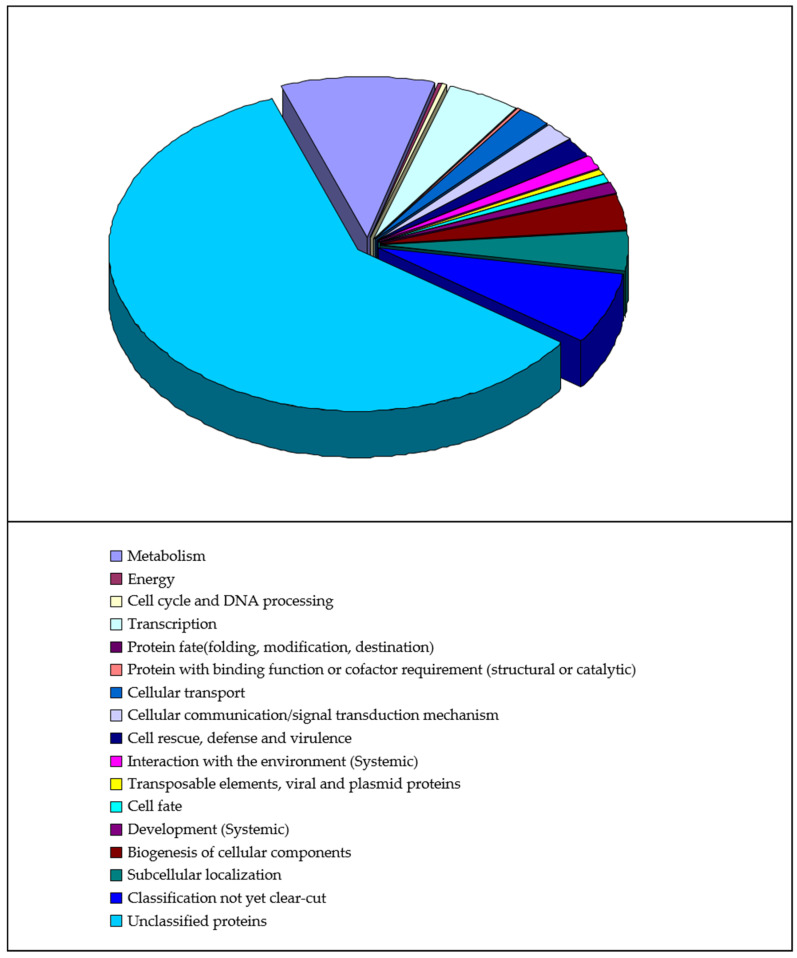
General biological process categorization on putative auxin-responsive genes. The sets of putative genes predicted in our study were analysed with the use of the MIPS from http://mips.gsf.de/ (accessed on 10 November 2021).

**Figure 3:**
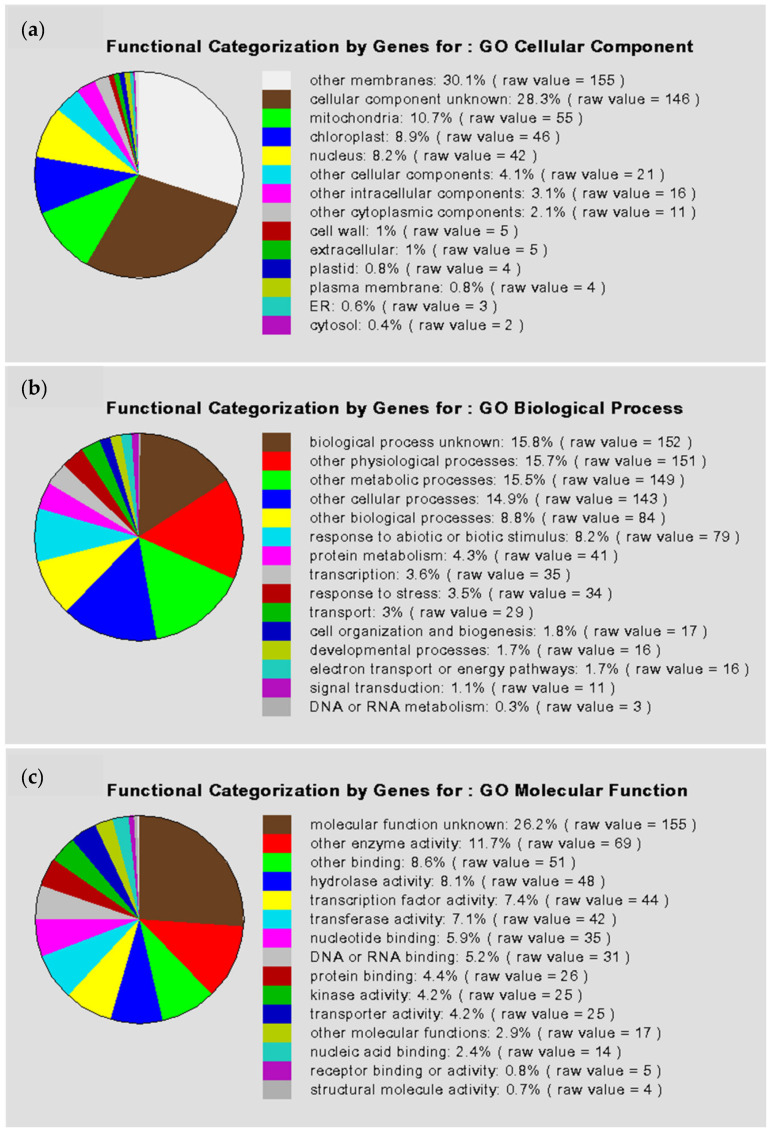
Pie charts for genes having GO functional annotation. The percentages were calculated with respect to the number of all redundant genes with annotation in each GO term. (**a**) GO terms of cellular component; (**b**) GO terms of biological process; (**c**) GO terms of molecular function.

All 474 putative auxin-responsive genes predicted in our study are distributed in a wide range of biological process classifications, as shown in Figure 2. Except for the unclassified proteins, the four largest categories are classification not yet clear-cut, metabolism, transcription, and subcellular localization (equal to biogenesis of cellular components). Moreover, the results of Gene Ontology (GO) data mining for independent candidate auxin-responsive genes (to exclude possible overlapping or redundant sequences) are shown in Figure 3. This result indicated that a large number of gene regulatory activities may have occurred following auxin treatment.

## 4. Discussion

It is of great importance and significance to identify the genes that respond to certain environmental hormone stimuli in plants because of the hormone’s vital role in regulating the course of most major developments and in maintaining plant physiological function. One strategy for annotating genomes and the discovery of target genes is to utilize the established knowledge of ortholog genes that have been experimentally verified and the conservation of ORFs of the genes across different species [27]. However, this approach is limited because, thus far, it has not been effective in the genome-wide discovery of many genes with specific functions. In addition, the approach is inappropriate for the identification of target genes that express under particular conditions, such as hormone reactions. As gene expression is basically regulated at the transcriptional level, the locations where TFs interact with *cis*-acting elements in the upstream promoter region of genes plays a significant role [28].

Our approach is complementary to traditional methods of gene discovery and functional annotation, which depend on the conservation of ORFs. This proposal of the *cis*-element-based method of targeted gene discovery uses the transcriptional regulatory information and is based on the following assumptions that motif modules of core AuxREs sites, greatly and regularly conserved because of their functional importance.

This study represents the computational discovery of a set of auxin response genes, which are regulated by a related element of AuxRE participation. Based on the most well-studied auxin reaction process, hormones regulate gene transcription through ARF, which specifically binds to these AuxREs. Therefore, we believe that the preservative AuxRE segment framework in promoter regions is an ideal indicator of a target gene’s response to auxin. Similar methods were applied and confirmed by previous work in identifying the putative genes of *A. thaliana* that are responsive to abscisic acid, abiotic stress, sucrose, heat, reactive oxygen species, drought and ethylene [18,23,29]; the predictions were successful, based on their evaluations of auxin-responsive genes by searching public gene expression databases and the literature. Our results further confirmed and extended the effectiveness of the *cis*-element-based targeted gene finding method, which generally has a high rate of prediction accuracy and is suitable for different species or/and various types of genes.

Obviously, the bioinformatics approach has advantages in detecting functional motifs on a genome scale [30].Computational methodology has been used in a genome-wide screening for *cis*-regulatory-element-based targeted gene discovery [18,23,29,31,32]; whereas, our study updates and differs from the previous work in the following important aspects. First, we have proposed an economical and efficient calculation method to predict a subclass of auxin response genes that combined the methods in recent similar studies [18,23] with the powerful computer programme, PRECISE (Prediction of Regulatory *CIS*-acting Elements), in the prediction of regulatory elements. Our method aims at detecting functional target genes on a genome scale with minimal false predictions. Second, most of the investigated public databases provided the rich experimental datasets and were uniform in the conventional naming of the genes. In addition, the spectrum of such genes from the public microarray/omics database is broad, and the genes are highly representative. Third, the promoters of verified auxin-responsive genes (excluding pseudo-genes and RNA genes) used in our study are more accurate than all genes (including pseudo-genes and RNA genes) for AuxRE prediction in *A. thaliana*.

## 5. Conclusions

Experiments have generated only a limited number of plant genes with well-characterized function. It is still unknown how many genes respond to exogenous auxin treatment. An economical and effective method was proposed for the genome-wide discovery of genes responsive to auxin in the model plant *A. thaliana*. Our method relies on *cis*-regulatory-element-based targeted gene finding across different promoters in its genome. Of note, as it is possible that other different types of *cis*-elements co-work with AuxRE to affect various regulations, our method could identify only a subclass of auxin-responsive genes because this approach cannot discover AuxRE-independent genes reacting to auxin through other TFs. With regularly updated information becoming increasingly available, including the information in databases, we expect this useful method to be widely applied in the future to various problems related to targeted gene finding. We believe that these sets of candidate auxin-responsive genes can significantly decrease the amount of identified underlying auxin target genes. In addition, the results provide useful functional clues for the annotating of genes that lack functional information.

## Figures and Tables

**Table 1:** Results of prediction evaluation of auxin-responsive genes based on computational methods.

Gene Category	Number of Genes	Of Genes with Known Function Annotaion (%)	Of All Candidate Genes (%)
supported by literature	32	32/276 = 11.6	32/474 = 6.8
supported by public datasets	291	193/276 = 69.9	291/474 = 61.4
supported by literature or public datasets	309	211/276 = 76.4	309/474 = 65.2
genes with known function annotation	276		
genes with unknown function annotation	198		
total number of candidates	474		

## Data Availability

All data is included in this paper.

## Abbreviations

AuxREs	auxin response elements
ORFs	open reading frames
TFs	transcription factors
ARFs	auxin response factors
GO	Gene Ontology
TSSs	transcription start sites
